# Development and Initial Validation of the Humor Climate in Sport Scale

**DOI:** 10.3389/fpsyg.2021.692892

**Published:** 2021-07-30

**Authors:** Gaute S. Schei, Tommy Haugen, Andreas Stenling, Anniken Grøtting, Derek M. Peters, Rune Høigaard

**Affiliations:** ^1^Department of Sports Science and Physical Education, University of Agder, Kristiansand, Norway; ^2^Department of Psychology, Umeå University, Umeå, Sweden; ^3^Department of Education and Sports Science, University of Stavanger, Stavanger, Norway; ^4^School of Allied Health and Community, University of Worcester, Worcester, United Kingdom

**Keywords:** humor, humor climate, sport teams, measurement, communication, group dynamics

## Abstract

In sport teams, humor is an essential element that influences communication processes, and plays an important role in group dynamics. Despite this, no current instrument is presented in the literature to measure humor climate in sport teams. Therefore, the current study presents the development and initial validation of the Humor Climate in Sport Scale (HCSS). The aim was to assess content, structural and concurrent validity of the developed instrument, and to examine differential item functioning (DIF) as a function of sex. Three different phases were completed in this study. The first phase involved focus groups (*n* = 5) that explored humor as communication in a team sport context. In phase 2, information from the focus groups was used to create a pool of potential items for the questionnaire. Two discussion groups with sport science students contributed to the development of 80 potential items, that two different expert groups then assessed for item quality. The final version of the instrument after this phase contained 14 items, representing three different humor dimensions. In phase 3, two independent samples with a total number of 776 participants were recruited for the psychometric evaluation of the instrument. EFA, ICM-CFA, and ESEM analysis were performed, supporting a three-factor structure with positive humor, negative humor in-group, and negative humor out-group. In addition, partial DIF as a function of sex on the negative humor dimensions was found, indicating differences in how male and female interpret the negative humor items. The findings in the current study expand our understanding of humor in sport teams and may be a starting point for further research on humor climate in sport teams and its role in group function.

## Introduction

Humor is an important element in communication between people and may influence interpersonal relationships and subsequently affect group processes and performance (Meyer, 2000; Caird and Martin, 2014). Humor has been conceptualized as a multifaceted construct that includes communication that others perceive as funny or makes someone laugh, mental processes producing, and perceiving amusing communication stimulus and the emotional satisfaction of it (Sliter et al., 2017; Martin and Ford, 2018). Research on humor in organizational psychology has a long tradition, and humor has been identified as a central factor affecting team interaction processes among leaders, managers, and employees (Avolio et al., 1999; Robert and Wilbanks, 2012; Lehmann-Willenbrock and Allen, 2014). In the sports context, research on humor is relatively sparse and has primarily focused on coaches’ use of humor (Grisaffe et al., 2003; Ronglan and Aggerholm, 2014). Considering the importance of intra-team communication in sport team functioning (and their subsequent performance), research investigating humor as part of communication within such sports teams is clearly warranted.

Theoretically the conceptualization of humor or the “sense of humor” considers humor as a cognitive ability (e.g., ability to generate mirth in others and to recognize and appreciate funny things that others say and do), a consistent behavioral pattern (e.g., people who joke and laugh, and always attempt to have fun), or as an emotion related trait (e.g., as a coping strategy to endure difficult situations) (Martin and Lefcourt, 1983; Thorson and Powell, 1993; Craik et al., 1996; Scheel and Gockel, 2017). These diverse conceptualizations of humor that emphasize its dispositional antecedents have muddied the distinction between “having a sense of humor” and “engaging in humorous communication,” and the consequences associated with these events (Sliter et al., 2017). Despite this, consequences of humor have been found to create an open atmosphere by awakening positive emotions that enhance listening, understanding, and acceptance of messages (Meyer, 1997; Greatbatch and Clark, 2002). Furthermore, humor has been related to less burnout (Abel and Maxwell, 2002), higher satisfaction (Decker, 1987; Booth-Butterfield et al., 2007), work-place creativity, and group cohesion (Romero and Pescosolido, 2008). Humor has also been found to buffer the stressor-strain relationship (Sliter et al., 2014), improve motivation, increase subsequent performance, and develop and maintain team culture (Clouse and Spurgeon, 1995; Avolio et al., 1999; Romero and Cruthirds, 2006; Guenter et al., 2013).

One of the most established frameworks for exploring humor is the dispositional humor styles model proposed by Martin et al. (2003). They conceptualized humor styles according to whether a person tends to prefer humor that enhances the self (intrapersonal) or relationships (interpersonal/social), and whether the humor is intended or perceived as being either positive or negative in nature. This created the following four humor styles that an individual may perceive: affiliative (interpersonal; positive), self-enhancing (intrapersonal; positive), aggressive (interpersonal; negative), and self-defeating (intrapersonal; negative). Based on this conceptual model, the four factor Humor Styles Questionnaire (Martin et al., 2003) was developed. Although several studies have demonstrated its reliability and validity (Kuiper and McHale, 2009; Romero and Arendt, 2011), some studies have reported inconsistent internal factor structure (Ruch and Heintz, 2016). For example, Sullivan and Dithurbide (2007) found little support for the original four-factor structure and concluded that a two-factor solution of positive humor (composite of affiliative and self-enhancing categories) and negative humor (composite of aggressive and self-defeating categories) had the best psychometric properties.

Drawing from both emotional contagion theory (Barsade, 2002; Hatfield et al., 2009) and the wheel model of humor (Robert and Wilbanks, 2012), these inherently personal humor styles when expressed within a social group may converge creating a relatively homogeneous humor “climate” within the group (Martin and Ford, 2018). Subsequently, we suggest that groups differ in combined levels of either positive or negative forms of humor depending on the most dominant humor styles present within the group members that contribute to the group’s overall humor climate (Kuiper and McHale, 2009; Robert and Wilbanks, 2012; Cann et al., 2014). Humor climate in an organizational context has been defined as: “a shared perception of how humor is used and expressed within an employee group” (Blanchard et al., 2014, p. 54). Blanchard et al. (2014) and Cann et al. (2014) have shown that in addition to being either positive or negative, humor can be focused inwardly toward members of the group or outwardly toward others outside the group. For example, Blanchard et al. (2014) investigated three dimensions of humor climate consisting of positive humor, negative in-group humor, and negative out-group humor. Their division of negative humor climate into two different dimensions explains how negative humor can have either beneficial or damaging consequences for the group. When the humor climate in the team is perceived as positive, regardless of whether it is targeting someone or something in-group or out-group, it will be able to strengthen the group. This assumption is in line with previous research indicating that positive humor is beneficial for team functioning, especially when the team is dealing with stressful situations or intra-team conflicts (Norrick and Spitz, 2008; Mesmer-Magnus et al., 2012). In contrast, a negative humor climate may be detrimental and have potentially dysfunctional consequences for individuals (e.g., reduced satisfaction and wellbeing; Kuiper and McHale, 2009), and groups (e.g., reduced cohesion and increased conflicts; Wood et al., 2007). The distinction between negative in-group and negative out-group humor may however be of great importance and nuance these findings. If the negative humor targets out-group members it may potentially have some positive effects (Martineau, 1972; Cann et al., 2014). Previous research has shown that negative out-group humor is related to cohesion, solidarity, and team identity (Terrion and Ashforth, 2002; Gockel and Kerr, 2015; Thomae and Pina, 2015). According to Ferguson and Ford (2008), negative out-group humor can create positive distinctiveness and social comparisons to enhance social identity within their own group. Furthermore, Ferguson and Ford (2008) argue that negative out-group humor can promote aggressive dispositions toward the out-groups, which could be a strategy for demonstrating superiority and potentially gain a competitive advantage (Aggerholm and Ronglan, 2012).

To attempt to assess humor climate in organizational team contexts, Cann et al. (2014) developed “The humor climate questionnaire” (HCQ). The HCQ assesses positive humor, negative humor (out-group and in-group), and in addition supervisor support for humor in the workplace. In Cann et al.’s (2014) study, after controlling for individual differences in humor style, the HCQ accounted for significant variance in several global and specific indicators of job experiences, including satisfaction. They found that positive humor explained more variance in relation to job satisfaction and commitment than did the presence of negative humor, and that the supervisor’s support for humor was generally a positive factor, predicting global satisfaction and positive aspects of organizational commitment. Out-group humor, on the other hand, was associated with dissatisfaction and lowered commitment to the organization. In Blanchard et al.’s (2014) study they also found that humor climate plays a role in how employees interpret ambiguous events within an organizational context and found it to affect their identification with the organization.

Despite the clear importance of humor and the humor climate in teams, there has been little research undertaken in team sport contexts. In sport research, humor has primarily been investigated in relation to the coaches’ use of humor (Grisaffe et al., 2003; Ronglan and Aggerholm, 2013, 2014; Høigaard et al., 2017), or humor as a personal attribute (Edwards and Jones, 2018; Kim et al., 2020). Høigaard et al. (2017) found that coaches’ use of humor predicted team identity, and Grisaffe et al. (2003) found that coach humor increased the athletes’ appreciation of the coach. In Ronglan and Aggerholm’s (2014) study, Scandinavian elite sport coaches interpreted and applied their humor as a conscious and integral part of their coaching practice, both for developing group and individual performance and for creating closeness between players and themselves. In a more recent study, Kim et al. (2020) investigated the nature of “team comedians” in sport. The study explored how team comedians act, develop, and influence other team members and the whole group. Their findings indicated that humor can be an important factor in team sport, contributing to positive outcomes like team integration, less tension, and greater pleasure among team members, but also that negative humor can hamper team functions.

Sex-differences in use and preference of humor have been given considerable attention over the years, with potential genetic (Schermer et al., 2017) and social (Robert and Wilbanks, 2012) explanations for the differences. From a sociological perspective, there are indications that males and females hold different appreciation and preferences of humor (Kuipers, 2015). According to Scheel and Gockel (2017) males tend to express and enjoy higher amount of aggressive and maladaptive forms of humor than their female counterpart. However, when examining sex differences in humor it is important to ensure that the instrument can capture true differences in the construct. Hence, psychometric analyses are needed to ensure that differences observed between males and females represent true differences in humor and not measurement non-invariance.

Although the HCQ represented an important step in advancing our understanding of humor climate in an organizational context, the HCQ is not directly applicable for the investigation of humor climate in team sport due to the lack of context in its item wording. There is a dearth of research investigating humor as a feature of interpersonal relationships in sport teams (Ronglan and Aggerholm, 2013; Sullivan, 2013), possibly because no sport-specific questionnaire for assessing humor climate has been developed. The main aim of this study therefore was to develop a measure of humor climate in sport teams and examine its psychometric properties. More specifically, we aimed to assess content validity, structural validity, and concurrent validity of the developed instrument, and examine differential item functioning (DIF) as a function of sex.

## Materials and Methods

Ethical approval was obtained from the Norwegian Social Sciences Data Service and by the Ethical Committee at the first authors’ University. This study includes three different phases in developing an instrument to measure humor climate in team sport: (1) focus group interviews to investigate humor climate theory in a sport specific context; (2) item generation; and (3) initial validation of the instrument. The first phase was designed to garner an understanding of how team sport athletes and coaches perceived the concept of humor in sport. In Phase 2, the participants’ expressions of their perceptions of humor were used in the development of possible items for the new questionnaire within the existing theoretical framework. In addition, the content validity of these items was examined by expert groups (researchers and former athletes). Phase 3 was concerned with item analysis (i.e., exploratory factor analysis) and subsequent confirmatory tests of the best fitting model (i.e., confirmatory factor analyses, exploratory structural equation modeling). We also examined a part of the nomological network surrounding the construct of humor climate by investigating relations between humor climate and social cohesion and social conflict. The protocol and results for these three phases are outlined in detail in the subsequent sections.

### Phase One—Exploring Humor as Communication in a Team Sport Context

Initially to explore humor in team sports, five focus group interviews were conducted, where the purpose was to capture how athletes and coaches experienced humor in their current and former teams, and how humor is perceived within sport teams. Subsequently we were also interested in getting an in-depth insight into the mental and emotional responses of mirth involved with humor. Twenty-one athletes (11 males and 10 females, range 17–31 years old) and five coaches (four males and one female, range 50–56 years old) contributed. Participants represented different team sports (e.g., handball, football, volleyball, ice-hockey, and rhythmic gymnastics). The focus group interviews for the athletes were organized due to their team affiliation. Group 1; six female elite athletes, Group 2: five male elite athletes, Group 3: four female junior elite athletes, Group 4: six male junior elite athletes, Group 5: This group consist of coaches with different team affiliation and sports with elite and junior-elite experiences.

Each focus group was moderated by a trained researcher and followed a standard semi-structured interview format (Longhurst, 2003) with (1) warm up session with introductory questions, (2) question around the following three main themes; (a) What is humor in team sport and what type of humor is prevalent in team sport, (b) How they perceived their own humor use and how they perceive coaches’ and teammates’ use of humor, (c) How they perceived the effect of various types of humor in relation themself (e.g., self-esteem, motivation, satisfaction, enjoyment) the team as a whole (e.g., intra-team communication, cohesion, conflict) and performance, and finally (3) ending wrap-up questions. During the focus group a poster was put forward on a table in the middle of the participants for each theme, and the participants was asked to talk freely around the themes. Participants were encouraged to share experiences from their former and current teams and were reminded that there were no “wrong or right answers.” The focus groups lasted an average of 50 min (range 44–60 min), were audiotaped and were transcribed verbatim into NVivo software (QSR International, Burlington, United States) for qualitative data analysis. Participants were given the opportunity to read through the transcribed material from their focus group interview and make necessary changes to the transcribed material if necessary. No participants wanted to read through the material, and the transcripts were approved for analysis. The transcribed material was analyzed using thematic analysis according to Braun and Clarke (2006). Initially the interviews were thoroughly read through searching for meaning, patterns, similarities, and inequalities, looking for factors that could describe humor as a form of communication in sport teams. The main interest was humor as a part of intra-team communication in sport teams with an investigation of different forms of humor and how they are expressed. Inductive and deductive approaches were utilized in analyzing and organizing the data. More specifically, humor theory from organizations (Blanchard et al., 2014; Cann et al., 2014) formed a deductive foundation in developing dimensions, while an inductive approach was used categorizing and understanding responses in a sport specific context (Fereday and Muir-Cochrane, 2006). A total of 32 codes that represented statements about humor in sport teams were organized into three main dimensions to establish a foundation for subsequent questionnaire item generation.

(1) *Positive humor*: Characterized as lighthearted humor originating from comical situations or histories, funny mistakes, practical jokes, and teasing that creates a positive atmosphere in the group. This was defined as positive humor including friendly, non-threatening humor that individuals share within their group.

*“We tease each other a lot, but it’s not in a bad way”. (Female volleyball player).*

*“You put a plastic glass of water under the helmet (ice-hockey), so when he takes out his helmet, he gets water all over him”. (Male ice-hockey player).*

(2) *Negative humor in-group*: Characterized as aggressive humor directed toward someone or something in-group that creates primarily a negative atmosphere for the in-group. This was defined as aggressive humor in-group originating from superiority, aggression, bullying or denigration.

*“Many can have fun, but on the behalf of one or two others. And I experience that as a negative type of humor, even though there are ten players laughing.” (Male handball coach).*

*“Yes, there are some players that have quit because of that, but if you play bad, and in addition gets a lot of banter, then it ends like that.” (Male football player).*

(3) *Negative humor out-group*: Characterized as aggressive humor that is directed toward someone or something out-group, that may create either a positive atmosphere or a negative atmosphere for the in-group. Defined as negative humor directed toward someone outside the group, originated by amusing banter, mocking, storytelling, or superiority.

*“In tournaments, players from other teams often have to be the referee, and some of them are so bad, and that is so funny so then we have a lot of fun with that.” (Female volleyball player).*

*“We talk a lot about dicks and ladies, and that kind of things. Same with sexuality, it’s easier to use that kind of insult when we have this aggressive humor.” (Male ice-hockey player).*

### Phase Two—Item Generation and Content Validity

The main aim of this phase of the study was to use the information gathered in phase one to create a pool of potential items for use in the questionnaire (Eys et al., 2009). Two 45-min open discussions with sport students at the first author’s university were conducted. In the first discussion group 11 sport science students (Master level) participated, and in the second discussion group 48 first-year sport science students participated. The participants were given a brief introduction about humor and the dimensions identified in phase one and subsequently produced items for positive and negative humor. In total these two discussion groups produced 80 items. An expert panel consisting of two professors, one associate professor, and one Ph.D. student organized the generated items into the main theorized and empirically investigated humor dimensions depending on their relevance for each dimension. This expert panel was familiar with the context, had comprehensive theoretical knowledge about the phenomenon, and broad experience of scale development. These experts examined each of the 80 items based on criteria as clarity of item wording, conciseness, grammar, reading level, face validity, and redundancy. Additionally, each item was assessed for relevance for athletes, accuracy, and similarity (Eys et al., 2009; DeVellis, 2017). Duplicates were removed in this process. Each investigator independently analyzed each item and recommended necessary changes. Unanimous agreement between the researchers was mandatory to keep an item. Potential disagreements were solved through discussions. The result of this process resulted in a pool of 40 items.

The remaining 40 items were then rated by a panel of five former athletes. These former athletes came from different team sports (ice hockey, handball, and football), and all five had competed at the highest level in their country. Three had experience from playing on the national team, and international clubs at the highest level. At the time, three of the participants worked in clubs at the highest level in Norway in different roles (e.g., coaches, administration). This panel of former players individually received information about the concept of humor climate, and the process of phase 1 developing humor dimensions. They were instructed to inspect all 40 items and make comments on each item. They evaluated the clarity and conciseness of the items and were also asked to identify any other items they could think of that would enable us to better explore the phenomenon of humor climate in team sport contexts (DeVellis, 2017). In addition, length, difficulty level, potential double-barreled items and ambiguity were evaluated (DeVellis, 2017). After input from these former players, the item pool was reduced to 15 items, containing five items on each of the three dimensions. Some remaining items were modified for clarity through this process. Last, to further assess content validity, the final items were critically examined by the expert panel. One item^1^ was in this process excluded from the instrument. It was hypothesized that this item could be ambiguous because the wording could be perceived as both positive and negative. Thus, the final version of the instrument consisted of 14 items in total, as shown in Table 1. Items were then placed in a questionnaire format (Table 1), with the stem “In my team”, attached with a Likert scale ranging from 1 (*strongly disagree*) to 7 (*strongly agree*) after each of the 14 items. Higher scores reflect stronger perceptions of either positive or negative humor (see Appendix Table A1 for the Norwegian version).

**TABLE 1:** Items in the HCSS.

In my team
PH1: Players do funny things
PH2: Players make fun of each other (joking, imitation, comments, tomfoolery)
PH3: Players tell funny jokes that make others smile and laugh
PH4: I experience friendly irony
NHI1: Players tell negative stories about each other to be funny
NHI2: Humor makes some players feel belittled
NHI3: The humor is characterized/tinged by discriminatory content
NHI4: Players and coaches use negative humor about each other to be funny
NHI5: Offensive humor is used about players
NHO1: People outside the team are imitated in a disrespectful way (support staff, players on other teams, referees, supporters, journalists)
NHO2: Malicious humor is used toward people outside the team (support staff, players on other teams, referees, supporters, journalists)
NHO3: Players use offensive humor about people outside the team (support staff, players on other teams, referees, supporters, journalists)
NHO4: Players laugh at disciminatory comments made about people outside the team (support staff, players on other teams, referees, supporters, journalists)
NHO5: Players use hostile humor about people outside the team (support staff, players on other teams, referees, supporters, journalists)

*PH, Positive humor; NHI, Negative humor in-group; NHO, Negative humor out-group. Response on Likert scale with 1 = Strongly disagree to 7 = Strongly agree.*

### Phase Three—Initial Validation of the Instrument

#### Participants

Two independent samples were recruited for the psychometric evaluation of the instrument. The first sample served as the primary exploratory sample, and the second sample was used to confirm the most appropriate model (DeVellis, 2017). Sample one consisted of 441 active handball (*n* = 295) and ice hockey (*n* = 146) players (180 female and 261 male, *M* age = 21.99, *SD* = 4.29, range 16–39 years). Participants came from 19 handball teams and 9 ice hockey teams, and 14 of the teams competed at the highest level in Norway, whereas the remaining 14 teams played in the second highest division. Teams were located in seven different counties in Norway. Participants had played for their team for *M* year = 2.86, *SD* = 2.43, Min = 1, Max = 16. Sample two consisted of 335 active football (*n* = 221) and handball (*n* = 114) players (193 female and 142 male, *M* age = 20.99, *SD* = 4.41, range 16–44 years). Participants were recruited from 14 football teams and 9 handball teams. Teams were competing in division three (*n* = 10), four (*n* = 9), and five (*n* = 4). Teams were located in two counties in Norway. Participants had played for their team for *M* year = 2.18, *SD* = 2.04, Min = 1, Max = 18.

#### Procedure

For the first data collection (sample one), 31 clubs were contacted and asked to take part in the study, three clubs declined to participate for different reasons (e.g., primarily lack of time). Three researchers visited 28 different clubs over a period of 5 months. For the second data collection (sample two), 29 clubs were contacted and asked to participate, and 23 clubs agreed to take part. Three researchers visited these clubs over a period of 3 months. The procedures were equal for both data collections. The purpose of the study was described to the whole team, and each player was provided with a letter of information and a consent form to be signed. Participants were informed they could withdraw from the study at any given time. Players received the questionnaire after giving their consent. Information was gathered through a hard copy questionnaire, containing questions about their team and their own individual characteristics, described in the previous Participants section. They completed the questionnaire before or after a training session or a match, depending on the conditions of each individual club. It took approximately 10–15 min to complete the questionnaire. Participants were guaranteed anonymity and confidentiality, and they were invited to contact researchers for a copy of the general results when the study was finished.

#### Measures

As a part of testing the nomological network (Cronbach and Meehl, 1955) of the newly developed HCSS-scale, we also sought to investigate the concurrent validity of the scale, based on associations with social cohesion (i.e., group integration social) and social group-conflict. A positive humor climate has previously been associated with beneficial group outcomes like cohesion and reduced conflict (Romero and Cruthirds, 2006; Blanchard et al., 2014). In contrast, a negative in-group climate has been argued to be detrimental for group functioning (Wood et al., 2007; Romero and Arendt, 2011). Negative humor out-group, however, has been found to be associated with both beneficial and detrimental outcomes within groups (Romero and Cruthirds, 2006; Cruthirds et al., 2013). Thus, as a test of the concurrent validity, we hypothesized that (a) positive humor climate would be positively correlated with social cohesion and negatively correlated with social conflict; and (b) negative in-group humor climate would correlate negatively with social cohesion and positively with social conflict. Based on the conflicting findings from previous research on negative humor out-group, we were not able to establish an *a priori* hypothesis regarding the relationship between negative humor out-group, social cohesion, and social conflict.

##### Social Cohesion

One subcomponent of the four cohesion-dimensions from the Norwegian version (Haugen et al., 2021) of the Group Environmental Questionnaire (GEQ; Carron et al., 1985; Eys et al., 2007) was used to collect data on social cohesion. Group integration social (GIS) was measured with four items. The participants responded to the items on a 9-point Likert scale with 1 (*strongly disagree*) to 9 (*strongly agree*). Higher scores reflect perceptions of stronger social cohesion.

##### Social Conflict

One dimension from the Norwegian version (Haugen et al., unpublished) of the Group Conflict Questionnaire (GCQ; Paradis et al., 2014) was used to assess social conflict (GCS). Participants responded to seven items on a 9-point Likert scale with 1 (*strongly disagree*) to 9 (*strongly agree*). Higher scores reflect perceptions of more social intra-group conflict.

#### Statistical Analyses

Mplus (Muthén and Muthén, 2017) version 8.4 was used to estimate the models with the full information maximum likelihood robust estimator (MLR), which provide standard errors and a chi-square test statistic that are robust to non-normality. Item-level missing data were accounted for by the MLR (Enders, 2010). Because the chi-square test of exact fit is sensitive to sample size and minor model misspecifications (Marsh et al., 2005), model fit was evaluated using several goodness-of-fit indices and criteria; the Tucker Lewis index (TLI) > 0.90, comparative fit index (CFI) > 0.90, root mean square error of approximation (RMSEA) < 0.08, and the standardized root mean square residual (SRMR) < 0.08 (Marsh, 2007).

In sample 1, Exploratory Factor Analysis (EFA) was carried out to assess the underlying factor structure and potentially refine the item pool. The EFA was applied with oblique Geomin factor rotation. A unique factor would only be considered if at least three items loaded onto a distinct factor. Items that exceeded an *a priori* criteria of factor loading at 0.400 and above and without substantial (>0.300) cross-loadings onto other factors were retained.

In sample 2, in line with recommendations in the literature (Marsh et al., 2013), both Independent Cluster Model Confirmatory Factor Analysis (ICM-CFA) and Exploratory Structural Equation Modeling (ESEM) were used to evaluate the EFA-informed best fitting hypothesized model of the HCSS-scale. When relying solely on ICM-CFA to examine the factor structure of a multidimensional scale, the factor correlations may be inflated due to the highly restrictive nature of the model specification (Marsh et al., 2014). ESEM may reduce some of the problems with ICM-CFA because it allows for the inclusion of cross-loadings between items and non-target factors. Instruments may include cross-loadings that can be justified by substantive theory, item content, or simply represent another source of measurement error. Thus, the items may be fallible indicators of constructs and tend to have small residual associations with other constructs (Asparouhov and Muthén, 2009). As most items have multiple determinants, it is reasonable to assume that most psychological measurements include non-zero cross-loadings (Marsh et al., 2014). Further, previous research shows that forcing cross-loadings to be zero may result in inflated factor correlations that undermine discriminant validity and lead to biased estimates (Marsh et al., 2013). The ESEM was estimated using oblique Target rotation with cross-loadings specified to be close to zero, but not exactly zero.

Because the participants in the present study were recruited from different teams, we accounted for the nested data structure by adjusting the standard errors and goodness-of-fit model testing using Muthen and Satorra’s (1995) aggregated analysis (i.e., TYPE = COMPLEX in Mplus).

A multiple indicator multiple causes (MIMIC) approach (Morin et al., 2016) was used to examine differential item functioning (DIF) as a function of sex. Compared to multi-group measurement invariance testing, the MIMIC approach is a more parsimonious approach that suits the relatively small sample in the current study. In line with recommendations in the literature (Morin et al., 2013, 2016), three models were estimated and compared: (i) a null effect model, in which all paths from the predictor to the latent variables and item responses were constrained to zero; (ii) a factors-only model, where the paths from the predictor to the latent variables, but not the item responses, were freely estimated; (iii) a saturated model, where the paths from the predictor to the item responses, but not the latent factors, were freely estimated. DIF is present if the saturated model provides a better model fit compared to the factors-only model. An improved model fit in the factors-only and saturated models compared to the null effects model indicate relations between the predictor and the ratings.

For the nested model comparisons, a CFI difference of less than 0.010 and RMSEA difference of less than 0.015 between the two models were considered evidence of equivalent fit to the data (Chen, 2007). The CFI was used as the main criterion because it is less sensitive to sample size and model complexity. Composite reliability was computed according to McDonald’s (1970)ω = (*Σ| λi|)^2^/([Σ| λi| ^2^]* + *Σδii*) using standardized parameter estimates from the ICM-CFA or ESEM models where *λi* are the factor loadings and *δii* are the error variances. McDonald’s omega coefficient can be interpreted similar as coefficient alpha, but do not rely on the tau-equivalence assumption (McNeish, 2018).

## Results

Table 2 presents descriptive statistics for the items in sample 1 and sample 2. Overall, observed means were relatively high (i.e., above 5.0 on a 7-point scale), compared to the numerical mean of the scale, for the positive loaded items. Similarly, observed means for negatively loaded items were relatively low (i.e., below 3.0 on a 7-point scale), except two items [NHI1 = 4.27 (sample 1) and NHI4 = 3.09 (sample 1)]. The skewness values ranged from −2.32 (PH2, sample 1) to 6.08 (NHO4, sample 2), and kurtosis values ranged from −1.12 (NIH1, sample 1) to 8.27 (NHO5, sample 2).

**TABLE 2:** Descriptive Statistics of the Items of the HCSS (Top Part-sample 1, Bottom Part-sample 2).

	*M*	*SD*	Skewness	Kurtosis	Range	*n*
**Sample 1**						
PH1	6.21	1.28	–1.75	3.49	1–7	441
PH2	6.43	0.88	–2.32	6.75	1–7	440
PH3	6.32	1.05	–1.89	4.05	1–7	438
PH4	6.08	1.27	–1.48	2.36	1–7	436
NHI1	4.27	3.52	–0.10	–1.12	1–7	434
NHI2	2.84	2.49	0.82	–0.18	1–7	434
NHI3	2.28	2.38	1.19	0.60	1–7	432
NHI4	3.09	3.09	0.57	–0.64	1–7	431
NHI5	2.19	2.12	1.32	1.08	1–7	433
NHO1	2.79	2.87	0.70	–0.46	1–7	440
NHO2	2.56	2.58	1.03	0.41	1–7	438
NHO3	2.89	2.94	0.72	–0.40	1–7	439
NHO4	2.60	2.78	0.87	–0.17	1–7	439
NHO5	2.46	2.65	1.03	0.19	1–7	438
**Sample 2**						
PH1	5.87	1.26	–0.75	–0.29	3–7	335
PH2	6.08	1.11	–1.08	0.53	3–7	333
PH3	5.92	1.28	–0.97	0.68	1–7	335
PH4	5.85	1.19	–0.88	0.59	1–7	334
NHI1	2.29	1.90	0.97	0.23	1–7	332
NHI2	1.88	1.51	1.74	3.34	1–7	333
NHI3	1.56	1.20	2.60	7.43	1–7	333
NHI4	1.61	0.98	1.99	4.49	1–7	334
NHI5	1.45	0.82	2.51	6.57	1–6	334
NHO1	1.77	1.19	1.79	3.94	1–7	333
NHO2	1.57	0.97	2.35	6.73	1–7	333
NHO3	1.63	0.99	2.12	5.71	1–7	333
NHO4	1.59	1.12	6.08	2.61	1–7	333
NHO5	1.51	0.94	2.61	8.27	1–7	332

*PH, Positive humor; NHI, Negative humor in-group; NHO, Negative humor out-group.*

As can be seen in Table 3, the three-factor solution yielded a better model-fit compared to the one- and two-factor solution. As shown in Table 4, the three-factor solution mirrored the hypothesized factor structure, with only negligible cross-loadings across factors. Factor one comprised the hypothesized positive humor items (factor loading range = 0.685–0.803), factor two comprised the hypothesized negative in-group humor items (factor loading range = 0.636–0.774), and factor three comprised the hypothesized negative out-group humor items (factor loading range 0.541–0.859). Although the estimated four-factor model revealed the best model fit, one of the factors consisted of only one substantial indicator (NHI1), with a factor loading of 2.203, providing additional support for the three-factor solution (Jöreskog, 1999). The three-factor model was thus retained as the final model from the EFA because it yielded a more interpretable factor structure than the other solutions. Composite reliability of the three factors in sample 1 was 0.849 (PH), 0.857 (NHI), and 0.896 (NHO).

**TABLE 3:** Goodness of Fit Statistics and Information Criteria for the EFA on the HCSS (sample 1).

	χ^2^	*df*	CFI	TLI	RMSEA	RMSEA 90% CI	SRMR	AIC	BIC
One-factor solution	939.926*	77	0.670	0.610	0.159	[0.150, 0.169]	0.145	19,904	20,076
Two-factor solution	381.145*	64	0.879	0.827	0.106	[0.096, 0.116]	0.056	19,187	19,412
Three-factor solution	155.979*	52	0.960	0.930	0.067	[0.055, 0.080]	0.025	18,917	19,191
Four-factor solution	96.092*	41	0.979	0.953	0.055	[0.041, 0.070]	0.020	18,874	19,192

df, Degrees of freedom; CFI, comparative fit index; TLI, Tucker-Lewis index; RMSEA, root mean square error of approximation; CI, confidence interval; SRMR, Standardized Root Mean Squared Residual; AIC, Akaike information criterion; BIC, Bayesian information criterion. EFA models were conducted with geomin oblique rotation, MLR estimator. *All χ^2^-values are significant (p < 0.001).

**TABLE 4:** Factor loadings for EFA (sample 1).

	One factor	Two factors		Three factors			Four factors			
	F1	F1	F2	F1	F2	F3	F1	F2	F3	F4
**Positive humor (PH)**										
PH1	0.033	0.756*	–0.006	0.755*	–0.037	0.021	–0.010	0.759*	–0.031	0.015
PH2	0.100*	0.799*	0.062	0.801*	0.059	0.001	0.016	0.793*	0.036	0.011
PH3	0.022	0.805*	–0.019	0.803*	0.003	–0.032	–0.018	0.813*	0.023	–0.047
PH4	0.037	0.684*	0.004	0.685*	–0.008	0.004	0.019	0.679*	–0.040	0.022
**Negative humor in-group (NHI)**										
NHI1	0.548*	0.227*	0.540*	0.241*	0.636*	–0.007	2.203	0.000	0.003	0.002
NHI2	0.555*	–0.011	0.557*	–0.003	0.774*	–0.104	0.046	–0.019	0.740*	−0.107*
NHI3	0.695*	0.004	0.695*	0.006	0.713*	0.096	–0.024	0.005	0.797*	0.022
NHI4	0.644*	0.071	0.642*	0.080	0.735*	0.017	0.064	0.055	0.664*	0.038
NHI5	0.751*	–0.020	0.753*	–0.016	0.659*	0.199*	–0.031	–0.016	0.759*	0.118
**Negative humor out-group (NHO)**										
NHO1	0.701*	–0.006	0.702*	–0.002	0.202*	0.541*	–0.006	–0.007	0.207	0.536*
NHO2	0.813*	–0.027	0.815*	–0.025	0.029	0.824*	–0.002	–0.030	0.039	0.819*
NHO3	0.711*	0.030	0.709*	0.033	–0.014	0.750*	0.036	0.021	–0.039	0.764*
NHO4	0.800*	0.027	0.798*	0.029	0.016	0.825*	–0.017	0.029	0.062	0.792*
NHO5	0.789*	–0.006	0.789*	–0.004	–0.030	0.859*	0.018	–0.011	–0.019	0.848*

*p < 0.05. Sample 1, N = 441.

*Boldface: Factor loadings on chosen factor solution.*

The results from the cross-validation in sample 2 are presented in Tables 5, 6. The three-factor ICM-CFA model yielded acceptable model fit, and all items had factor loadings larger than 0.568 on the intended factors. The three-factor ESEM analysis yielded slightly better model fit compared to the ICM-CFA model, with a CFI difference larger than 0.010 and RMSEA difference larger than 0.015 between the two models. In the ESEM, all items except one loaded acceptably on the target factor, with no substantial cross-loadings. The exception was item NHI1, which had a factor loading of 0.315 on the target factor (negative in-group) and 0.355 on the negative out-group-factor.

**TABLE 5:** Factor loadings for ICM-CFA and ESEM (sample 2).

	Three-factor ICM-CFA				Three-factor ESEM			
	PH	NHI	NHO	*δ*	PH	NHI	NHO	*δ*
PH1	0.772*			0.404*	0.775*	0.048	−0.015	0.404*
PH2	0.735*			0.460*	0.743*	0.001	0.052	0.446*
PH3	0.803*			0.355*	0.796*	−0.025	−0.042	0.358*
PH4	0.568*			0.677*	0.565*	−0.056	−0.075	0.660*
NHI1		0.603*		0.636*	0.104*	0.315*	0.355*	0.598*
NHI2		0.771*		0.405*	0.004	0.676*	0.106	0.422*
NHI3		0.816*		0.334*	0.024	0.757*	0.067	0.346*
NHI4		0.820*		0.327*	−0.042	0.953*	−0.150*	0.282*
NHI5		0.855*		0.268*	−0.033	0.915*	−0.063	0.242*
NHO1			0.705*	0.503*	0.025	0.042	0.673*	0.502*
NHO2			0.859*	0.262*	−0.052*	−0.005	0.868*	0.250*
NHO3			0.813*	0.340*	0.061	−0.062	0.869*	0.318*
NHO4			0.813*	0.339*	0.033	0.188	0.653*	0.348*
NHO5			0.882*	0.222*	0.012	0.048	0.844*	0.224*

Boldface: Factor loadings on intended factor, *p < 0.05. Sample 2, N = 335. δ = item uniquenesses (residual variances).

*PH, Positive humor; NHI, Negative humor in-group; NHO, Negative humor out-group.*

**TABLE 6:** Goodness of Fit Statistics and Information Criteria for the ICM-CFA, ESEM, and MIMIC Models (sample 2).

	χ^2^	*df*	CFI	TLI	RMSEA	RMSEA 90% CI	SRMR	AIC	BIC
ICM-CFA	145.141*	74	0.968	0.961	0.054	[0.041, 0.066]	0.050	11,363	11,534
ESEM	88.065*	52	0.984	0.972	0.046	[0.028, 0.062]	0.020	11,340	11,596
Null effects model	182.952*	66	0.952	0.924	0.073	[0.060, 0.085]	0.086	11,341	11,596
Factors-only model	131.395*	63	0.972	0.953	0.057	[0.043, 0.071]	0.027	11,290	11,557
Saturated model	85.942*	52	0.986	0.972	0.044	[0.027, 0.060]	0.019	11,264	11,573
Partial DIF^*a*^	100.408*	56	0.982	0.966	0.049	[0.033, 0.064]	0.033	11,272	11,566

*df, Degrees of freedom; CFI, comparative fit index; TLI, Tucker-Lewis index; RMSEA, root mean square error of approximation; CI, confidence interval; SRMR, Standardized Root Mean Squared Residual; AIC, Akaike information criterion; BIC, Bayesian information criterion.*

ICM-CFA model was conducted with target oblique rotation, ESEM model was conducted with target oblique rotation. MLR estimator. *All χ^2^ values are significant (p < 0.001). ^*a*^Sex included as predictor of the items of the two negative humor dimensions.

The latent factor correlations between the positive humor climate dimension and the two negative humor climate dimensions were relatively weak and not statistically significant in both the ICM-CFA model (NHI with PH: −0.099, *p* = 0.124; NHO with PH: −0.055, *p* = 0.473) and the ESEM model (NHI with PH: −0.076, *p* = 0.189; NHO with PH: −0.016, *p* = 0.812), whereas the latent factor correlation between the two negative humor climate dimensions were relatively strong (ICM-CFA: NHO with NHI: 0.799, *p* < 0.001 and ESEM: NHO with NHI: 0.772, *p* < 0.001). Composite reliability of the latent factors from the ICM-CFA model were 0.814 for PH, 0.888 for NHI, and 0.910 for NHO.

### Concurrent Validity

The measurement model of the four-item social cohesion (group integration social—GIS) subscale of the GEQ was excellent [χ^2^(*df* = 2, *N* = 333) = 1.847, *p* = 0.397; TLI = 1.000; CFI = 1.000; RMSEA < 0.001 (0.000–0.106); and SRMR = 0.009]. The initial measurement model of the seven-item social conflict subscale (GCS) of the GCQ yielded close-to-acceptable fit [χ^2^(*df* = 14, *N* = 333) = 54.152, *p* < 0.001; TLI = 0.896; CFI = 0.931; RMSEA = 0.093 (0.067–0.120); and SRMR = 0.043]. An inspection of modification indices revealed high covariance between two items; item 1 (“Personal friction among members of our team leads to angry confrontations at social gatherings”) had high covariance with item 2 (“The heated disagreements among members of our team in social situations become personal”). Allowing these two items to covary resulted in improved model-fit [χ^2^(*df* = 13, *N* = 333) = 36.988, *p* < 0.001; TLI = 0.933; CFI = 0.959; RMSEA = 0.074 (0.047–0.103); and SRMR = 0.033]. However, the re-specifications did not affect the interpretations of the latent variable correlations in the overall model. Due to the controversies surrounding *post hoc* modifications to improve model-fit (Hermida, 2015), we decided to proceed with the initial measurement model of the GCS. The composite reliability of the GIS and GCS was 0.894 and 0.916, respectively.

When testing the latent variable correlation between HCSS and GIS and GCS, the ICM-CFA model of the HCSS was assessed. The overall model comprising PH, NHI, NHO, GIS, and GCS yielded acceptable fit-indices [χ^2^(*df* = 264, *N* = 335) = 469.704, *p* < 0.001; TLI = 0.942; CFI = 0.949; RMSEA = 0.048 (0.041–0.055); and SRMR = 0.051]. The latent variable correlations are displayed in Table 7.

**TABLE 7:** Latent variable correlations (sample 2).

	PH	NHI	NHO	GIS
NHI	−0.103 [−0.229, 0.022]	–		
NHO	−0.058 [−0.208, 0.091]	0.798* [0.672, 0.942]	–	
GIS	0.382* [0.237, 0.528]	−0.096 [−0.260, 0.068]	−0.053 [−0.223, 0.117]	–
GCS	−0.173* [−0.301, −0.045]	0.511* [0.371, 0.650]	0.397* [0.236, 0.557]	0.069 [−0.084, 0.222]

Standardized correlation estimates and [95% CI]. * Statistically significant (p < 0.01).

*PH, Positive humor; NHI, Negative humor in-group; NHO, Negative humor out-group.*

As shown in Table 7, there was a positive correlation between positive humor climate and social cohesion, and a negative correlation between positive humor climate and social group-conflict. The two negative humor climate dimensions (in-group and out-group) were both positively associated with social group-conflict. In addition, the positive correlation between the two negative humor climates (in-group vs. out-group) remained relatively high.

Finally, model fit comparisons indicated that the saturated model had a better model fit than the factors-only model (ΔCFI = 0.014 and ΔRMSEA = 0.013), which suggest DIF as a function of sex (Table 6). Examination of the parameter estimates showed that females provided lower ratings than males on the items of the two negative humor dimensions (Table 8). Thus, we estimated a partial DIF model where sex predicted the items of the two negative humor dimensions (but not the positive humor items), which provided a comparable level of fit as the saturated model (Table 6). Taken together, these results suggest partial DIF as a function of sex, indicating that females provided lower ratings than males on the negative humor dimension items.

**TABLE 8:** Effect of sex on the item responses in the saturated MIMIC model (sample 2).

	β	*b*	*SE*	*p*
**Positive humor**				
PH1: Players do funny things	0.019	0.044	0.147	0.764
PH2: Players make fun of each other (joking, imitation, comments, tomfoolery)	–0.123	–0.263	0.163	0.106
PH3: Players tell funny jokes that make others smile and laugh	–0.136	–0.311	0.171	0.069
PH4: I experience friendly irony	–0.081	–0.178	0.116	0.126
**Negative humor in-group**				
NHI1: Players tell negative stories about each other to be funny	–0.339	–0.946	0.132	<0.001
NHI2: Humor makes some players feel belittled	–0.166	–0.413	0.188	0.028
NHI3: The humor is characterized/tinged by discriminatory content	–0.307	–0.680	0.179	<0.001
NHI4: Players and coaches use negative humor about each other to be funny	–0.289	–0.576	0.137	<0.001
NHI5: Offensive humor is used about players	–0.312	–0.573	0.150	<0.001
**Negative humor out-group**				
NHO1: People outside the team are imitated in a disrespectful way	–0.300	–0.663	0.105	<0.001
NHO2: Malicious humor is used toward people outside the team	–0.309	–0.616	0.111	<0.001
NHO3: Players use offensive humor about people outside the team	–0.234	–0.472	0.112	<0.001
NHO4: Players laugh at disciminatory comments made about people outside the team	–0.363	–0.776	0.121	<0.001
NHO5: Players use hostile humor about people outside the team	–0.328	–0.642	0.121	<0.001

*Sex coded as 0 = male, 1 = female. β, standardized regression coefficient; b, unstandardized regression coefficient; SE, standard error; p, p-value.*

## Discussion

The aim of this study was to develop and examine the psychometric properties of a humor climate scale for use in team sport. Humor has been identified as an important phenomenon in sport contexts that may influence interpersonal relationships and team functioning. Humor climate has the potential to both strengthen various group processes or be detrimental to individuals in sport teams. Still, lacking a suitable questionnaire to assess humor climate, little research has been conducted to explore these relationships. The HCSS provides a measure that has the potential to fill this gap and offers an appropriate tool to conduct more research on this essential phenomenon. The overall scale development process was based on recommendations from previous research by Eys et al. (2009) and DeVellis (2017) and allowed us to develop the 14-item HCSS that measures three dimensions of humor climate: positive humor, negative humor in-group, and negative humor out-group.

First, to establish content validity, a literature review, focus group interviews, discussion groups, expert reviews, and revisions by former elite athletes were conducted in line with suggestions from DeVellis (2017). These steps were performed to optimize the instrument’s content in relation with the humor climate construct we wanted to measure (Johnson and Morgan, 2016). With an extensive procedure exploring humor as communication in a team sport context and through item generation, content validity of the HCSS was supported.

The three-factor ICM-CFA model of the HCSS demonstrated satisfactory results with acceptable model fit with strong factor loadings. The three-factor ESEM model also demonstrated acceptable results with a marginally better model fit on the CFI and the RMSEA. Despite a slightly improved model fit on the ESEM model, the ICM-CFA provides more parsimony to our final model. Kline (2016) argues that a proposed model provides support for the interpretation if the instruments’ validity when the items targeting a certain factor have high factor loadings, and when correlations between factors are not overly high. In the ICM-CFA model, factor loadings ranged from 0.568 (PH4) to 0.882 (NHO5), providing acceptable factor loadings on the intended factors. The latent factor correlations between positive humor and negative humor in-group, and between positive humor and negative humor out-group were weak and non-significant. Previous studies have reported non-significant correlations (Curseu and Fodor, 2016) and low-to-moderate correlations (Martin et al., 2003; Blanchard et al., 2014; Cann et al., 2014) between positive humor and negative/aggressive humor. Thus, our findings in relation to correlation between positive humor and negative humor mirror previous research, and the lack of association indicates that positive and negative humor can be seen as two distinct facets of humor (Martin et al., 2003). The latent factor correlation between negative humor out-group and negative humor in-group was, however, quite strong (ICM-CFA: 0.799, ESEM: 0.772), indicating a relatively high degree of shared variance, and thus, possibly low discriminant validity. According to Blanchard et al. (2014), an overlap between negative humor out-group and in-group is to be expected as the two factors have substantial conceptual similarities that may explain this overlap. Although this correlation is considerably higher than the correlation between positive humor and negative humor in-group and negative humor out-group, each correlation is below 0.90 and the factors are identified as unique factors (Kline, 2016). Overall, the factor analysis from the ICM-CFA and ESEM support the three-dimensional structure of the HCSS.

Even though there are no established cut-offs regarding the magnitude of target factor loadings and cross-loadings in ESEM models, some guidelines have recently been provided (Morin et al., 2020). Cross-loadings below 0.300 could be considered negligible, whereas cross-loadings larger than 0.300 should be inspected further. The cross-loadings in the present study were mostly negligible. However, one item identified in the EFA in sample 1 as negative humor in-group factor (NHI1: Players tell negative stories about each other to be funny) had a substantial cross-loading on negative humor out-group (0.355) in the ESEM model in sample 2. One explanation could be that for this item, the “out-group” target is perceived somewhat differently. Negative humor directed toward a teammate who is physically present (as was the intended meaning of the item) should be considered as in-group humor. However, if negative humor expressions directed toward a teammate occur when he or she is not present (i.e., backbiting), the same item may be perceived as out-group humor. Further exploration of this item should be conducted, and this substantial cross-loading should be scrutinized in future studies. Still, this item captures an important feature of negative humor climate in sport teams that the expert groups in phase two regarded as important, thus supporting the inclusion of this item in the questionnaire.

In terms of concurrent validity, we tested the relation between the humor dimensions and group integration social (GIS) and social conflict (GCS). Positive humor was, as expected, positively related to GIS and negatively related to GCS. This is consistent with previous humor research (Romero and Pescosolido, 2008), and our results (see Table 7) contribute to further confirmation of this relation. This indicates that our conceptualization of positive humor in the HCSS appears both theoretically and conceptually meaningful.

Negative humor has previously been connected to both cohesion and conflict (Meyer, 2000; Cruthirds et al., 2013), and former research is divergent on what kind of outcomes that are related to negative humor out-group (Romero and Cruthirds, 2006; Cann et al., 2014; Scheel and Gockel, 2017). Our results showed negative humor in-group had a statistically significant relation with GCS, but not a statistically significant relationship with GIS. Negative humor out-group was also examined in relation to GIS and GCS. The results revealed a significant relation between negative humor out-group with GCS. The similar relations between negative humor in- and out-group with GIS and GCS may be explained by a previously hypothesized distinction between aggressive humor and mild aggressive humor (Romero and Cruthirds, 2006), where the intended meaning is to communicate a forceful message with a humorous pitch, but it is interpreted as aggressive humor, increasing levels of conflict. This differentiation is not accounted for in the HCSS and this may explain why negative humor out-group might be experienced as fun and joyful in a team, but still consist of maladaptive content, and therefore in some cases lead to conflict (Ronglan and Aggerholm, 2013). Future studies are therefore required to assess how different levels of “aggressiveness” in negative humor affect humor climate within teams. Lastly, negative humor out-group may have other characteristics that we were not able to capture in the HCSS and these may be related to other group variables that are important in team sports. No previous studies have managed to separate positive and negative humor climate in team sports, but our initial findings seem promising.

When testing the nomological network, the latent variable correlations supported the hypotheses that positive humor is positively connected to cohesion, and negatively connected to conflict. Furthermore, greater negative humor (both in-group and out-group) was related to greater social conflict. Previous studies have established the beneficial impact positive humor can have on different group processes and group outcomes in organizations (Romero and Pescosolido, 2008; Mesmer-Magnus et al., 2012), and have also highlighted the potential destructive effects of negative humor (Wood et al., 2007). Findings in the present study reflect these relations and therefore support the usefulness of this construct in team sport settings. Investigating the nomological validity of the HCSS contributes to establishing repercussions of certain degrees of different humor climate within sport teams. Previous studies on humor climate lack investigation of nomological validity (Cann et al., 2014; Curseu and Fodor, 2016), and consequently it is difficult to compare our results of nomological validity with previous humor climate studies. Despite this, we argue that the CFA results support the nomological validity of our theoretical relations, and we encourage other researchers to examine these further. We suggest that further exploration of the nomological network includes personal dispositions such as extraversion, agreeableness, and neuroticism (Zillig et al., 2002; Hüffmeier and Hertel, 2011). To further investigate concurrent validity in relation to humor climate, we also encourage researchers to examine other factors that theoretically should be related to humor climate. Relevant factors to investigate may be the relation between the HCSS subscales and individual humor styles, other group factors that are central in sport teams, and the way in which coaches/leaders may affect the humor climate in their team.

Humor scales are commonly assumed to measure the same attributes for both males and females (Blanchard et al., 2014; Cann et al., 2014). Studies regarding sex differences in humor usually depend on mean comparisons estimated by humor scales (Martin et al., 2003). However, the mean differences in humor can be attributed to a true difference, measurement bias, or a combination of both. Thus, insufficient evidence of absence of measurement bias compromises the conclusions made on group-comparisons.

Our analyses revealed partial DIF as a function of sex (scalar non-invariance), indicating that females provided lower ratings than males on the negative humor-climate items. A superior model fit for the saturated model indicates that the sex-differences in the present study are driven by differences at item-level, not on latent constructs level. The sex-difference at item-level indicates that males and females perceive and interpret the items differently. Specific words and sentences used in the negative humor dimensions can leave room for ambiguity that may render the interpretation of its intended meaning. Thus, social, biological, and cultural differences between the sexes may be responsible for differential response patterns toward negative humor climate and therefore elicit biased responses to items. Although not completely explainable, one could argue that as a general trend, offensive, discriminatory, and negative humor are more socially acceptable among males in a masculine culture (Martin et al., 2003; Wood et al., 2007), and that this manifests into how the items are perceived. In contrast, typical female characteristics such as empathy and sensitivity may lead to the perception that negative humor behavior are undesirable, causing the females to underreport their engagement in such behaviors.

## Conclusion, Future Research, Limitations

The aim of this study was to develop and examine the psychometric properties of an instrument to measure humor climate in sport teams. We have advanced our understanding of the construct of humor climate in a new context and provided researchers with an instrument to assess humor climate in team sport contexts. First, our findings support a division of humor climate into three different dimensions: positive humor, negative humor in-group, and negative humor out-group. Second, our results revealed two different dimensions of negative humor, supporting previous research (Cruthirds et al., 2013). Moreover, our study supports research indicating that negative humor in-group may have a stronger negative effect on group processes, than negative humor directed outwards (Blanchard et al., 2014; Scheel and Gockel, 2017), confirming that knowing both the direction and the style of humor is vitally important (Romero and Cruthirds, 2006; Cann et al., 2014). Third, our newly developed instrument demonstrated statistically significant correlations between humor climate and group integration social and social conflict. These relations are of interest in further investigations, and future research could also examine other constructs of group dynamics in relation to humor climate. Lastly, participants responded to the Norwegian version of the HCSS questionnaire. There is a need to examine the psychometric properties of the English, and indeed any other language versions of the HCSS in future research.

This study has several strengths that contribute to a significant addition to humor research. This is the first study assessing humor climate in a team sport context, and comprehensive work has been completed to optimize the quality of the HCSS. No previous study on humor in sport has recruited a sample size with the magnitude in this present study, including athletes from three different team sports. We also consider it a strength that females and males are represented, including both elite and sub-elite athletes. Second, items were grounded with a foundation from previous research, qualitative interviews, discussion groups, and lastly expert revision. In addition, solid statistical analysis including EFA, ICM-CFA and ESEM has been conducted and we have provided complete transparency in our process of developing items for, and creation of the final version of the HCSS.

There are, of course, some limitations that are important to mention. First, the present study only examined a limited set of validity (structural, concurrent) and reliability aspects of the HCSS and additional tests of validity (e.g., predictive) and reliability (e.g., ICC) are warranted. Second, one item (NHI1) loaded significantly on both negative humor in-group and out-group in the ESEM model. Even if we argue that this item can be difficult for athletes to separate if the target is in-group or out-group, it was included in the questionnaire based on the results from the ICM-CFA model and support from the expert group. Nevertheless, this item should be explored in further research and comprehensively examined to more fully understand how it contributes to team sports humor climate.

Because of practical issues, clubs varied when they filled out the questionnaire during different weeks in the season. Therefore, conditions like tiredness, stress, time of day, may influence how players responded to the questionnaire. Whether this factor is considered a confounder remains unclear. Moreover, the present study does not investigate the temporal stability (test-retest reliability) of the developed scale. We encourage future studies to incorporate this when further examining the instrument. Lastly, future studies would do well to develop the scale to other languages and examine the scale in different cultural- and sport contexts. In conclusion, our study supports the construct validity of the HCSS and we encourage further examination of its psychometric properties in other samples, contexts and cultures. Particularly, the practical impact of the scalar non-invariance according to sex identified in the present study require further investigation. Future studies should aim for larger sample sizes and even distribution between groups to ensure rigorous multi-group tests of measurement invariance in this (and other) humor scales.

## Data Availability Statement

The raw data supporting the conclusions of this article will be made available by the authors, upon request.

## Ethics Statement

The studies involving human participants were reviewed and approved by the Ethical Committee of the Faculty of Health and Sport Science, University of Agder. Written informed consent to participate in this study was provided by the participants.

## Author Contributions

GS, TH, and RH contributed to the conceptualization, development, data curation, former analysis, and the writing of the manuscript. AS contributed to statistical analyses and writing of the manuscript. AG contributed to data curation and reviewing of the manuscript. DP contributed to the writing of the manuscript and feedback on the conceptualization of the study. All authors contributed to manuscript revisions and approved the submitted version.

## Conflict of Interest

The authors declare that the research was conducted in the absence of any commercial or financial relationships that could be construed as a potential conflict of interest.

## Publisher’s Note

All claims expressed in this article are solely those of the authors and do not necessarily represent those of their affiliated organizations, or those of the publisher, the editors and the reviewers. Any product that may be evaluated in this article, or claim that may be made by its manufacturer, is not guaranteed or endorsed by the publisher.

## Footnotes

1 “Players laugh of their own mistakes.” Considered imprecise and ambiguous, because sometimes it may be ok (training), but not at other times (competitions). Also: which mistakes are we talking about? Which mistakes are acceptable to make fun of (in a competitive context) and when is it acceptable?

## Appendix

**TABLE A1:** Norwegian version of the HCSS.

I mitt lag.
PH1: Finner spillere på humoristiske påfunn
PH2: Tuller spillere med hverandre (vitser, imitasjon, kommentarer, narrestreker)
PH3: Forteller spillere morsomme vitser som skaper smil og latter
PH4: Opplever jeg vennlig ironi
NHI1: Forteller spillere negative historier om hverandre for å vaere morsom
NHI2: Er humoren slik at enkelte føler seg mindre
NHI3: Er humoren preget av diskriminerende innhold
NHI4: Bruker spillere og trenere negativ humor om hverandre for å vaere morsom
NHI5: Blir humor om medspillere brukt på en krenkende måte
NHO1: Imiteres personer utenfor laget på en respektløs måte (støtteapparat, spillere på andre lag, dommere, supportere, journalister)
NHO2: Brukes ondsinnet humor om personer i idrettsmiljøet (støtteapparat, spillere på andre lag, dommere, supportere, journalister)
NHO3: Bruker spillere støtende humor om personer utenfor laget (støtteapparat, spillere på andre lag, dommere, supportere, journalister)
NHO4: Ler spillere av diskriminerende kommentarer om personer utenfor laget (støtteapparat, spillere på andre lag, dommere, supportere, journalister)
NHO5: Bruker spillere fiendtlig humor om personer utenfor laget (støtteapparat, spillere på andre lag, dommere, supportere, journalister)

*PH, Positiv humor; NHI, Negativ humor inn-gruppe; NHO, Negativ humor ut-gruppe. Svar på Likert skala med 1 = Helt uenig til 7 = helt enig.*
